# Uptake contexts and perceived impacts of HIV testing and counselling among adults in East and Southern Africa: A meta-ethnographic review

**DOI:** 10.1371/journal.pone.0170588

**Published:** 2017-02-16

**Authors:** T. Charles Witzel, Wezzie Lora, Shelley Lees, Nicola Desmond

**Affiliations:** 1 Sigma Research, Department of Public Health and Policy, London School of Hygiene and Tropical Medicine. London, United Kingdom; 2 Malawi-Liverpool Wellcome Trust Clinical Research Programme. Liverpool School of Tropical Medicine. Blantyre, Malawi; 3 Department of Public Health and Policy, London School of Hygiene and Tropical Medicine. London, United Kingdom; 4 Department of International Public Health, Liverpool School of Tropical Medicine, Liverpool, United Kingdom; Boston University, UNITED STATES

## Abstract

**Introduction:**

HIV testing and counselling (HTC) interventions are key to controlling the HIV epidemic in East and Southern Africa where HTC is primarily delivered through voluntary counselling and testing (VCT), provider initiated testing and counselling (PITC), and home-based counselling and testing (HBVCT). Decision making processes around uptake of HTC models must be taken into account when designing new interventions. Counselling in HTC aims to reduce post-test risk taking behaviour and to link individuals to care but its efficacy is unclear. This meta-ethnography aims to understand the contexts of HTC uptake in East and Southern Africa and to analyse the perceived impacts of counselling-based interventions in relation to sexual behaviour and linkage to care.

**Methods:**

We conducted a systematic literature review of studies investigating HTC in East and Southern Africa from 2003 –April 2014. The search and additional snowballing identified 20 studies that fit our selection criteria. These studies were synthesised through a thematic framework analysis.

**Results:**

Twenty qualitative and mixed-methods studies examining impacts of HTC models in East and Southern Africa were meta-synthesised. VCT decisions were made individually while HBVCT decisions were located in family and community units. PITC was associated with coercion from healthcare providers. Low quality counselling components and multiple-intersecting barriers faced by individuals mean that counselling in HTC was not perceived to be effective in reducing post-test risk behaviour and had limited perceived effect in facilitating linkage to care.

**Conclusion:**

HBVCT is associated with minimal stigma and should be considered as an area of priority. Counselling components in HTC interventions were effective in transmitting information about HIV and sexual risk, but were perceived as ineffective in addressing the broader personal circumstances preventing sexual behaviour change and modulating access to care.

## Introduction

HIV testing and counselling (HTC) interventions have long been a cornerstone of efforts to slow the HIV epidemic, with various models of service delivery in operation that have a key goal of reducing the burden of undiagnosed infections [1]. As East and Southern Africa have been particularly affected by the HIV epidemic [2], there has been a significant drive to expand testing within this region in order to reach a wider population and slow the spread of HIV [3].

Based on growing consensus that those on successful antiretroviral therapy are unlikely to transmit HIV, control of the HIV epidemic in sub-Saharan Africa (SSA) is now moving towards a test and treat approach, signified by raising the threshold for HIV treatment from 350 to 500 CD4 per cubic millilitre [4–6]. Treatment as prevention (TasP) approaches to controlling the epidemic are key to the HIV strategies of several countries in SSA, with national efforts focused on rolling out and optimising new HTC programmes in an effort to improve clinical and behavioural outcomes [2,7].

There are various models for delivering HTC to different populations including voluntary client-initiated counselling and testing (VCT), provider-initiated testing and counselling (PITC), and home-based HIV testing and counselling (HBVCT) [8]. VCT takes place in a clinical facility and emphasises the need for pre and post-test counselling as well as the provision of informed consent by the individual being tested [9]. PITC operates under an opt-out approach and is targeted as part of routine care, at pregnant women, or at individuals with illness associated with HIV [9]. HBVCT is a form of VCT delivered in the community, with the same emphasis on counselling and informed consent [1]. VCT and PITC target the needs of key-populations affected by the HIV epidemic, while home-based approaches in East and Southern Africa function largely as screening for the general population whilst ensuring access to hard-to-reach and marginalised groups not typically reached by VCT programmes, as well as men and women living in rural areas who are less likely to access health service-based testing.

Counselling has long been key in HIV testing interventions. Successive World Health Organisation (WHO) guidelines emphasised the role that counselling plays in reducing post-test risk behaviour through developing risk reduction strategies and linking individuals to care following a positive result, including the provision of support in navigating the psychological and social barriers to accessing treatment [10, 11]. The counselling components in HTC, however, can be of variable quality with different levels of focus depending on service aims and commissioning environment. There are also questions about the ability of counselling to address the goals attributed to it, with poor evidence that these goals relating to sexual behaviour change and linking to care are successful [9]. Further to this, critics argue that these aspirations are inappropriate and unrealistic for a brief intervention of this type, leading to questions as to whether the purpose of the counselling element in HTC should be reframed as an intervention simply providing information and boosting motivation to reduce sexual risk and link into care [9, 12]. At a global policy level this shift is in progress, with the WHO’s 2015 HIV testing guidelines highlighting the role of counselling in HTC as primarily motivational [13]. While encouraging, changes in practice will likely take a significant amount of time to filter down.

A recent meta-ethnography provides insights into the factors enabling and deterring HTC uptake in SSA including lay constructions of risk, mental and social burden of living with HIV, gender inequality and health service features [14]. While there is systematic evidence exploring the impact of VCT on sexual behaviour [15, 16], and uptake of support following PITC [17], evidence is mixed as to the ability of HTC interventions to meet the short and long-term behavioural goals ascribed to their counselling components. In one systematic review on changes in risk behaviour following VCT, statistically significant differences in condom use were seen only amongst those testing HIV positive [16]. In a further review, those undergoing PITC demonstrated increases in condom usage and testing frequency following exposure to the intervention [17]. A recent meta-ethnography focusing on barriers to patient retention in care in SSA did not identify counselling as a key facilitator [18]. The experiences of individuals undergoing HTC through different models is therefore vitally important to understand given the primacy that is afforded to behaviour change in these interventions [10, 11]. It is therefore imperative that HTC interventions are understood from the perspective of those who undergo them in order for lessons to be drawn as to how best to expand testing to meet the diverse needs of individuals in East and Southern Africa (ESA). Understanding the uptake context of these interventions can provide key information as to how best to expand testing depending on the needs of a population.

The primary aim of this meta-ethnographic review is to consolidate anthropological and qualitative understandings of the experiences of adults who have undergone HTC in East and Southern Africa. The objectives are to develop an understanding of the experiential context of HTC uptake for individuals by model and to understand the perceived impacts of these interventions in relation to sexual behaviour and linkage to care. By focusing on adults (those over the age of 18) such a review can provide a greater degree of insight into the specific needs of an adult population, without conflating specific issues related to adolescents, HIV and health service accessibility such as differing risk profiles, increased barriers to accessibility and differing constraints related to culture [19].

## Methods

### Search strategy and identification of papers

This meta-ethnography followed the approach outlined by Noblit and Hare [20]. This six-step framework provides a systematic approach to collecting and synthesizing evidence. In particular, this method involves the systematic searching for and gathering of data, followed by familiarisation with the selected studies, drawing relations between texts, and finally synthesizing. More detail about our approach is provided below.

The database search was carried out by the first author between January and April 2014. This was a key period in the publication of relevant manuscripts and we consider this search up to date. Databases included in the search were SCOPUS, PubMed, BASE, Web of Science, and the Cochrane library. Predefined keywords were grouped around HIV testing intervention type, and then combined with keywords reflecting research methods and geographical interest, and then with key terms reflecting thematic interests (see Table 1).

**Table 1:** List of keywords.

One of: HIV testing, HTC, VCT
AND a combination of: impacts, Africa, qualitative, mixed-methods
AND one of more of: gender based violence, link to care, risk behaviour, counselling, gender, intimate partner violence, perceptions, anthropology, sociology, AIDS.

Titles and abstracts were reviewed, and if it remained unclear whether the article met study inclusion criteria of being related to impacts of HIV testing and incorporating qualitative research, limited automated searching of full texts was carried out for key terms ‘HIV testing’, ‘Qualitative’ and ‘Interview’ (See Table 2). At this stage 2062 studies were screened, and 82 selected for full text eligibility screening [CW].

**Table 2:** Exclusion and inclusion criteria.

	Exclude	Include
**Publication Date**	Pre-2003	January 2003-April 2014
**Publication type**	Non-peer reviewed content	Peer reviewed studies
**Study design**	Quantitative surveys, studies with no qualitative element.	Qualitative research, ethnography, results from focus groups / interviews, mixed-methods studies.
**Study population**	Under 18s.	Adults 18 years and older.
**Report types**	Reviews, opinion pieces, letters to the editor style work	Peer-reviewed primary research, qualitative meta-synthesis
**Language**	Non-English language	English language
**Geographic area**	Non-ESA countries	East and Southern Africa
**Topic**	Non-HIV testing	Impacts of HIV testing

### Quality assessment

For eligibility screening, a quality and relevance assessment based on the COREQ and BMJ guidelines on rigour in qualitative studies was carried out in duplicate [21, 22] [CW & WL]. See supporting information S1 Table–Quality and relevance assessment for the quality and relevance assessment framework. For studies where there was no unanimous agreement consensus was reached through discussion.

**Fig 1:**
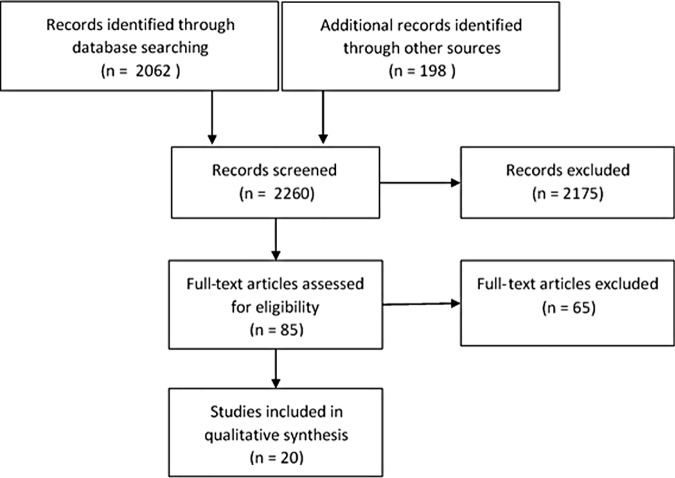
Literature search flow chart.

Of the 82 studies reviewed, 14 were agreed upon, but consensus was not immediately reached on 11 due mainly to differences in relevance scores. Of these, 5 were included and 6 discarded (2 because of low quality and 4 because they were deemed not to be relevant). Under the assumption that the most recent studies would contain the greatest number of relevant citations, we selected 5 studies for snowball sampling where we examined their reference lists for eligible studies. This led to a further search of 198 titles and abstracts [CW]. Of these 3 were deemed of interest and progressed to full text review, and 1 passed duplicate quality and relevance assessment and was included in the synthesis. See Fig 1 for details and Table 3 in results for included studies.

**Table 3:** Study summaries.

First author, year	Primary aim	ocation	Sampling Strategy	Sample	Data Collection	HTC Type
1. Bunnel et al, 2005 [23]	To identify challenges for discordance, challenges and prevention strategies.	Kampala, Uganda	Purposive sampling	67 individuals (32 women, 35 men)	In-depth interviews, focus groups	VCT
2. Bwambale, 2008 [24]	Determining the prevalence and factors associated with VCT use among men.	Bukonzo West district, Uganda	Cluster sampling	Quantitative: 780 Men	Quantitative: Survey	VCT
				Qualitative: 44 individuals	Qualitative: 4 Focus Group Discussions (FGDs)	
				Healthcare workers	10 Key Informant Interviews	
3. Daftary, 2007. [25]	Explore decision making process for HIV testing and serostatus disclosure by 21 patients hospitalised with M/XDR-TB.	Durban, South Africa	Not explicit	21 patients	21 In-depth interviews	VCT
4. Emusu, 2009 [26]	Gain a deeper understanding of the nature and contexts of the sexual violence experienced by women in HIV sero-discordant unions.	Kampala, Jinja, & Mbale Uganda	Not explicit	26 women	26 interviews using critical incident interviewing	VCT
5. Groves, 2010. [27]	Explore women’s experiences of consent during PITC.	Durban, South Africa	Purposive	25 women	25 semi-structured interviews	PITC
6. Jürgensen, 2013. [28]	To investigate possible explanations for high acceptance of HBVCT.	Monze, Southern Province Zambia	Quantitative: Cluster randomisation	Quantitative: 1694 in 36 clusters (18 clusters offered HB-VCT).	Quantitative–Baseline and follow up survey	HBVCT
			Qualitative: Purposive	Qualitative: 27 Men and women	Qualitative: 22 in-depth Interviews, 1 FGD	
7. Kyaddondo, 2010. [29]	Examine the experiences of HBVCT clients in relation to 1) the process of mobilisation, 2) counselling, consent, privacy and confidentiality, 3) disclosure, 4) referral to care	Kumi, Eastern Uganda	Cluster sampling	395 men and women	395 semi-structured questionnaires	HBVCT
8. Lifshay, 2009. [30]	To inform development of effective interventions for HIV-positive individuals.	Uganda (Jinja and two unidentified rural areas)	Purposive	48 HIV+ individuals	37 Interviews (10 excluded, 1 died)	VCT
9. Lubega, 2013. [31]	Explore reasons for continued attendance and attrition during ANC care for HIV positive women.	Iganga, Uganda	Not explicit	7 key Informants	20 In-depth interviews	PITC
				20 HIV+ women	7 Key informant interviews	
				112 carers and relatives	10 FGD with carers and relatives	
10. MacPherson, 2012. [32]	Better understand the patient, provider and health service barriers and facilitators contributing to progression through the care pathway.	Blantyre, Malawi	Purposive sampling from a cohort study	30 adults	40 Semi-structured interviews	VCT
				5 counsellors, 3 nurses, 2 clinical officers		
11. Musheke, 2013 [33]	Examine the experiences of couples participating in PITC.	Lusaka, Zambia	Maximum variation sampling	10 couples (3 discordant)	10 couple interviews	PITC
					7 individual interviews	
				5 women and 2 men abandoned by spouses	7 key informant interviews	
				5 lay counsellors and 2 nurses	Participant observation	
12. Nyanzi-Wakholi, 2009 [34]	Investigating the role of VCT and treatment in enabling HIV-positive Ugandans to cope with HIV.	Uganda	Purposive	108 participants from ECohort and DART trials	12 FGDs	VCT
13. Rohleder, 2005 [35]	Investigate the impact of the unclear positions of counsellors in a health service providing VCT.	Khayelitsha, South Africa	Convenience sampling	29 counsellors	16 Interviews	VCT
					3 FGD	
14. Rujumba, 2012 [36]	Exploring pregnant HIV positive and HIV negative women’s partner disclosure experiences and support needs.	Eastern Uganda	Purposive sampling from ANC testing	15 HIV+ and 15 HIV- women	30 in-depth interviews	PITC
				One doctor, two counsellors and three nurse midwives	6 key Informant interviews	
15. Sarna, 2009. [37]	To understand changes in sexual behaviour after treatment initiation and factors influencing condom use.	Mombasa, Kenya	Stratified purposive sampling	23 adults receiving ART	23 in-depth interviews	VCT
16. Sethosa, 2005 [38]	To evaluate HTC, self-disclosure, social support and sexual behaviour change among HIV reactive patients among a rural sample of HIV reactive patients.	Rural South Africa	Convenience sampling	55 HIV positive people.	55 semi-structured interviews	PITC
17. Shamu, 2010 [39]	Explore women’s and health workers’ perspectives and experiences of sexuality and sexual violence in pregnancy, including in relation to HTC.	Harare Zimbabwe	Not explicit	64 Pregnant / nursing mothers	7 FGDs	PITC
				7 Health Workers	7 Key informant interviews	
18. Sikasote, 2011 [40]	To understand the influence of VCT and an-HIV negative result on subsequent sexual behaviour and to identify the specific felt needs of those testing negative.	Copperbelt province, Zambia	Purposive Purposive	55 clients	42 initial interviews	VCT
				25 psychological counsellors	32 follow-up interviews	
					3 FGDs	
19. Siu, 2014 [41]	To explore the social context and relations that shape men’s access to HTC.	Busia district, Eastern Uganda	Purposive and snowball	26 men	26 in-depth interviews	VCT
20. Taegtmeyer, 2013 [42]	To understand Kenyan providers’ attitudes towards and experience with counselling MSM in a research clinic targeting this group for HIV prevention.	Coastal Kenya surrounding Mombasa	Convenience sampling	13 counsellors and 3 clinicians delivering HTC	16 Semi-structured in-depth interviews	VCT

### Analysis strategy

Studies were summarised using a standardised form detailing first author name, research date, location of research, sample, sampling strategy, data collection methods, intervention type, key themes, secondary themes, and recommendations [CW]. Primary and secondary themes were extracted from all studies.

Following extraction of primary and secondary themes, the lead author developed a thematic framework by forming adult nodes from primary themes with child nodes made up of secondary themes. This was then commented upon, tweaked and finally agreed upon within the study team, piloted on two studies and refined for conceptual clarity. Analysis was undertaken using QSR NVivo10 [CW].

Following broad coding using this framework, nodes were printed and hand coded into iterative codes. Final synthesis was performed by lifting quotations from iterative codes into a Microsoft Word document, separating themes into first (views of research participants) and second order constructs (the interpretation of research participants’ views). Third order constructs (the author’s synthesis of connections between first and second order constructs) were then produced through drawing linkages between constructs and analysing them inductively [CW]. See S2 Table titled Thematic construct table for an abridged version of the construct tables produced in this process. There is not a publicly available protocol for this review.

## Results

### Reasons for HTC uptake

In this section we examined the most common narratives concerning decision-making for individuals undergoing HTC (see Table 4 for full results). Following this, we explored four common barriers and facilitators to the uptake of testing and how they relate to each model.

**Table 4:** Context of HTC uptake.

Third order labels	Third order constructs	Second order constructs	Source material
**1. Contexts of HTC decision making vary by model.**	1.1 VCT as a response to threats to identities from enduring illness.	• Postponing engagement with VCT services until visibly unwell [34, 38, 41].	[34, 32, 38, 40, 41]
		• Fears that illnesses were visible to others [38].	
		• Individuals sought VCT when health failed sufficiently to mitigate fear around an HIV positive result [32].	
	1.2 VCT as a means to restore order in response to past risk(s)	• Individuals seek VCT after a risk event [38, 40].	[38, 40, 41]
		• Desire to regain control as key motivator for seeking VCT [40].	
		• Death of spouse or others in sexual networks prompts individual seeking VCT [41].	
	1.3 Encouragement from individuals in social or supportive networks in context of VCT and PITC	• Men discuss HIV testing mainly with peers. Involved reviewing sexual histories and risk discussions [41].	[27, 38, 41]
		• Women who felt they had a choice in testing for HIV had thought about it previously. Many had discussed testing with family or partners [27].	
	1.4 HBVCT decisions located in household and community spheres with pressure applied through social roles.	• Community leaders supported HBVCT interventions by encouraging individuals to test [28].	[28, 29]
		• Interactions and discussions among partners and other family members influenced individual decisions to test at home. [29].	
		• Three potential sources of influence on testing decisions were the partner, the headman and the counsellor [28].	
	1.5 Power dynamics within PITC in ANC coerce women into testing by relying upon their familial roles and obligations.	• There was little opportunity for pregnant women to refuse HTC as the health workers are thought of as powerful, senior members of the community [27].	[27, 28, 32, 33]
		• A dominant message that pregnant women received at the clinic is that being tested for HIV is the right thing to do for the health of the baby [27].	
		• Couples were deprived of the right to consent through health care workers evoking maternal/paternal responsibility to encourage uptake of HIV testing [32].	

#### Personal illness

The predominant theme around accessing VCT for both men and women focused on using testing as a diagnostic tool in the context of prolonged illness that threatened privileging aspects of identity (see construct 1.1 in Table 4). We conceptualise identity as how individuals perceive themselves and their social roles in a given social, historical and cultural context. VCT was perceived as the gateway to antiretroviral therapy [32, 38, 40, 41]. Access was heavily gendered with women seeking testing and treatment earlier than men in order to support their families and men seeking VCT once illness began to impact on their social and familial responsibilities, and social identities.

#### Risk perceptions

Utilising VCT to restore order in response to threats to identity that was threatened by perceptions of HIV risk was a significant theme amongst women who accessed VCT. These perceptions were often the result of feelings of vulnerability based on past sexual experiences, experiences of sexual abuse, or because of suspicions of marital infidelity and a resulting loss of trust [38, 40]. For men, and a limited number of women, this was sometimes framed in relation to behaviour while under the influence of alcohol or through an inability or unwillingness to control one’s own desires [40].

Testing in response to risk did not feature prominently in those accessing HBVCT and PITC services, where discourses focused on family and community dynamics rather than individual thought processes [28]. This is largely because these interventions were understood to be population wide screening efforts, and because those accessing VCT self-present to services.

#### Social interactions

Social interactions modulated individual decisions to test in a variety of ways. VCT was experienced as voluntary, while subtle pressure was applied through household and community decision making processes during HBVCT interventions. PITC was often not experienced as voluntary, with significant perceptions of coercion exerted by health workers in antenatal care (ANC) especially [27].

In the context of VCT, those in supportive networks sometimes encouraged others to test. This influence could be from health workers [38], or those in social networks such as communities more broadly, peers, families and the workplace [41]. Peer networks were important to men, encouraging friends to test, particularly if there was knowledge about past risk taking [41]. See construct 1.3 to 1.5 in Table 4.

In contrast, HBVCT locates HIV testing as a screening tool within the home, and individuals’ decision making around testing is framed within wider familial or community relationships (see construct 1.4). Indeed, testing decisions in HBVCT were often subject to subtle pressure from family members and through community leaders. Different aspects of individual social identities are mobilised in the application of pressure to test in various ways- through duty as a son or daughter, as a responsible community member and as a subservient wife [29]. Community and village leaders legitimise the process of testing through allowing access to HBVCT testing teams and encouraging others to test [28].

PITC was often not experienced as a choice by those offered it. PITC most often occurred in ANC where pregnant women felt that testing was ‘mandatory’ [27, 32]. Two dominant themes relating to coercion emerged within ANC which reflect this. The first is that power imbalances were exploited by clinical staff in coercing women to test [27, 33]. Health workers were powerful and important community members, and pregnant women often felt unable to refuse [32]. The second is that testing was the right thing to do for the baby [27]. Appealing to the women’s sense of duty as mothers, or as those who were about to become mothers, clinical staff in ANC successfully used coercive tactics to encourage women to test by framing it as for the health of the unborn child [32].

In the context of coercion, social identities were mobilised by individuals to meet a variety of personal and organisational aims around HIV testing. The decision to opt-out is not clearly articulated, and women’s additional role as (future) mothers is used as leverage by health care workers in insisting that testing is carried out. These testing decisions are framed less by biomedical need and more in terms of duties relating to social roles constraining agency and limiting the ability of women to opt-out.

### Barriers and facilitators to HTC

Multiple barriers and facilitators to each model of testing were identified. While it is beyond the scope of this review to explore them comprehensively, the four most important warrant attention: stigma, health service features, fear and gendered norms. These were all experienced differently as either barriers or facilitators and to a greater or lesser extent based on the type of testing intervention.

#### Stigma

Stigma has been recognised as a key barrier for individuals accessing HTC interventions, particularly in VCT settings [25, 28, 32]. Individuals were often reluctant to access testing because of fears around being seen at HIV clinics, as well as the social implications of a positive diagnosis [25, 28, 32]. HBVCT and PITC did not have significant levels of stigma associated with them and thus facilitated uptake.

#### Health service features

Health service features were important determinants of the likelihood that individuals would utilise VCT and PITC services. Some clinics operated at inconvenient times, or were not seen as sufficiently confidential [28]. In those who had tested, confidentiality, proximity and convenience were key features facilitating access to testing [24], all of which were described as facilitators in individuals who accessed HBVCT [28, 29].

#### Fear

Fear of a positive result was a significant feature in reluctance to test, in VCT and in PITC although to a lesser extent [24, 25]. This did not appear to be a significant barrier in HBVCT.

#### Gender norms

There were widespread differences in men and women seeking HIV testing. Based on traditional gender roles and their relationship to health seeking behaviour, men generally left testing later when unwell [28, 34]. Women were more likely to test sooner, particularly after noticing symptoms in children or sexual partners [34]. Traditional gender roles were a particular barrier in VCT, a facilitator in PITC and either in HBVCT depending on the context [28].

### Counselling and sexual behaviour

#### Counselling and motivation

A key goal of HTC interventions is to provide information on HIV and sexual health, and to help motivate individuals to take-up and sustain behaviour change. High quality counselling interventions seemed to achieve motivational aims and were effective in providing information on sexual health and condom use, which in turn led to increased perceptions of risk [39]. Counselling was effective to a degree in debasing myths around HIV and increasing knowledge about condoms and how to use them [39]. This motivation was not perceived to be sufficient to produce aspired outcomes in relation to behaviour change however.

#### Multiple, interrelated barriers to change

The overarching theme related to barriers to change was the inability of counselling to address broader patient circumstances relating to risk behaviour. This was underpinned by multiple and related sub-themes (see themes 2.1 to 2.6 in Table 5). Barriers to sexual behaviour change after HTC were experienced at several levels in multiple ways by individuals who had tested both positive and negative. These barriers were structural, cultural, related to preferences and to quality of care. These multiple levels intersected with multiple social roles, and cultural and economic contexts creating a conflicting and dynamic set of assumptions that individuals operate within. These were often operationalised in conjunction with each other depending on individual circumstances.

**Table 5:** Perceive impacts of HTC in relation to sexual behaviour and linkage to care.

Third order labels	Third order constructs	Second order constructs	Source material
**2. Counselling interventions are inadequate to meet their stated aims in relation to sexual behaviour change and linkage to care due to the convergence of multiple barriers.**	2.1 Inability of counselling to address broader patient circumstances affecting risk behaviour.	• VCT counsellor training was inadequate for working with MSM. Most learnt on the job [42].	[30, 32, 35, 42]
		• Respondents were only advised about condom use, not reducing partners. Women reported less support from counsellors, family and friends around reducing frequency of sex [30].	
	2.2 Sexual pleasure and linked condom preferences as barriers to risk reduction.	• Pain experienced by women when using condoms, continued sexual desire, partners’ desire for children, and assumptions about sero-concordance posed challenges for risk reduction [30].	[30, 33, 37, 42]
		• Many women and female partners disliked using condoms because they caused pain during intercourse [30].	
	2.3 Feelings of inevitability surrounding HIV transmission constraining choice.	• In long relationships between discordant partners, a sense of immunity coupled with a degree of fatalism obstructed condom use [37].	[26, 30, 37]
	2.4 Issues relating to gender roles within relationships modulated by socio economic factors.	• Suspicions of infidelity and blame around HIV infection shaped intimate partner violence. Being younger, physically weaker, and/or economically dependent on male partners hindered the women’s ability to resist sexual advances of partners perceived to be at high risk of transmitting or getting infected with HIV [26].	[26, 30, 33, 36, 38, 39, 40, 42]
		• Women’s financial dependence on their male spouses limited their ability to seek care after diagnosis [31].	
	2.5 Cultural beliefs about HIV and HIV transmission.	• Assumptions that if one partner tested negative the other must also be negative were common [39].	[24, 26, 32, 36, 37, 39, 40]
		• Sero-discordance was not believed to be possible [24].	
	2.6 Tensions between notions of safety and expectations based on traditional gender roles.	• Condoms were associated with casual sex. They were not believed to have a place in marital or long term sexual relationships [33].	[33, 37, 40]
		• Childbearing desires of both male and female respondents limited their ability to use condoms consistently [37].	
	2.7 Low quality interventions lead to patient attrition from care pathways.	• Post-test counselling provided to expectant mothers was inadequate [31].	[31, 32, 35]
		• Care was infantilising and created dependency [32].	

Significant ambivalence around the role of counselling in empowering individuals to adopt behaviour change was attributed to multiple factors. Participants tended to place blame on inadequate or patronising counselling [32] and lack of longer term post-test follow up [30]. Counsellors and healthcare workers tended to cite pressures of working in an under-resourced or task oriented health care system [35], and their own views about participants’ homosexuality [42]. Both groups identified the multiple and complex sets of personal circumstances individuals operate under (see constructs 2.2 to 2.6) [32, 33, 35, 37, 42] and the inability of counselling to address cultural beliefs about sero-discordance held by partners (construct 2.5) [24, 34, 39] as being key barriers to behaviour change. Counselling interventions were perceived as inadequate by both counsellors and participants, creating a significant source of frustration within the clinical encounter and limiting the positive impact of counselling interventions [32, 35, 42].

#### Linkage to care

Barriers and facilitators to entering HIV care are experienced at multiple levels including (but also beyond) those which affect the HTC intervention [32]. Health service factors, social and cultural influences and the circumstances of individuals are also key [28, 31, 32, 33].

The quality of counselling was raised as a major factor in patient attrition in the studies reviewed, particularly following provider initiated testing and counselling in ANC [31, 33]. This was frequently associated with issues surrounding working in under-resourced settings and a lack of training, both particularly pressing issues in ANC.

Social and cultural factors such as beliefs about HIV fatalism also influenced individuals’ ability to access care following an HIV diagnosis, and counselling did a poor job in countering these [26, 31, 37]. Furthermore, traditional constructions of masculinity often limited the accessibility of HIV testing services to men, which potentially also prevented men from engaging in care following a positive result [28, 41]. Counselling did not seem effective in countering these issues across the models studied.

Beyond counselling, individuals’ relationships were significant determinants of their ability to seek care, and lack of disclosure was a major barrier in accessing care across all models of testing [31, 33]. Some who had positive experiences of disclosure indicated that the supportive relationships that followed helped them to sustain ART [33].

The ability of those to seek and access care is therefore contingent on circumstances at multiple levels including health service features, social and cultural factors, and personal circumstances. While HTC interventions can help link to care, there are significant barriers external to the intervention that must be overcome in order to effectively reduce patient attrition.

## Discussion

### Context of HIV testing & implications for expansion

An important finding of this meta-ethnography is the way in which individuals experience the different models of HTC available to them, particularly relating to the four most common barriers and facilitators. VCT was generally used as a diagnostic tool when social identities were threatened by ill health, or to restore order in response to risk. There were very significant barriers related to accessing VCT, limiting its utility for much of the population who were not unwell or did not perceive themselves to be at risk of infection. The context in which VCT is accessed and experienced therefore indicates that focusing expansion of testing on VCT services will provide limited returns if the aspiration is to improve rates of testing generally. This is consistent with VCT’s place in these health systems as a diagnostic intervention reliant on self-presentation.

In comparison, HBVCT and PITC in ANC were experienced as screening interventions, and therefore had higher acceptability to the participants in these studies. Both intervention types had lower barriers to access than VCT, with HBVCT appearing to be particularly acceptable to those who accessed it. This is perhaps emphasised due to the epidemic context of the region. Because the HIV epidemic is generalised, and ones’ HIV status can be seen to be highly consequential for others, these interventions are effective because they are embedded within social relationships. The uptake context of these interventions and in particular the lack of stigma associated with HBVCT indicates that a broader roll-out of HBVCT may be effective in increasing HTC uptake in the region, particularly if targeting whole communities rather than individuals or ‘risk groups’. HBVCT has been shown to be effective in reducing disparities in testing rates between populations in urban and rural Zimbabwe [43], and it is conceivable that the same benefits could be seen across the rest of the region reaching a key goal of testing hard to reach populations through the expansion of HTC. Opt-out PITC as part of primary care or in hospital settings may also be highly acceptable and effective for the same reasons, but in this context it is essential that lessons are learned from the experience of women who are tested during ANC in order to avoid widespread coercion, which remains a critical issue with PITC [44].

Through understanding the utility of intervention type from the perspective of those undergoing testing, policy makers and commissioners can expand services in line with the needs of particular populations. Clearly in high prevalence areas, which lack VCT services, VCT and PITC are likely to be priorities. For areas with existing services which are being underutilised or which have high levels of undiagnosed HIV, HBVCT will likely be an effective method to screen individuals in a way that is highly acceptable. This may be particularly true for men, as testing provision in the region tends to focus on women, and because clinics can be understood as feminised spaces by the populations they serve [45]. HIV self-testing may be a particularly cost effective home-based strategy in reaching those under-represented in facility-based options [46]. Further, a key benefit of HBVCT is that those who are identified as positive tend to have higher CD4 counts than in other models so significant knock on costs to the health service and wider economy can be saved and further infections averted through early treatment [47], linking to the TasP agenda.

### Addressing the individual: Sexual behaviour & linkage to care

The results indicate that the role of counselling components within HTC should be reconceptualised. It is clear that the many goals ascribed to them [10, 11] are inappropriate for the type of intervention given the multiple and interrelated barriers that individuals face. While counselling components in testing were believed to be effective in transmitting information about HIV and HIV treatment across all models, there was significant ambivalence from participants and healthcare workers alike surrounding the ability of counselling to assist in practical risk reduction and in retention to care. The frustration that both counsellors and those who had undergone testing felt at the inability of counselling to help patients address the broader factors in their sexual risk taking, and the individual circumstances limiting their ability to access care need to be taken into account when designing new services.

While it is imperative that counselling quality is addressed, in particular with enhanced training and support, it is also clear that beyond disseminating messages surrounding HIV transmission the role of counselling in reducing risk behaviour is limited. This is largely because brief counselling interventions as part of HTC cannot deal with the broader social and structural determinants of sexual risk within a population characterised by high levels of economic dependency, high levels of HIV transmission occurring within marriages, constraints to agency, and additional personal factors such as preferences for and meanings surrounding condomless sex. While counselling did have a role in linking individuals to care, and indeed poor counselling led to patient attrition from the care cascade, the social and cultural context in which the HTC interventions were embedded meant that often individuals faced significant barriers which these interventions could not overcome. This is consistent with evidence exploring the multiple barriers to maintenance of ARV in the region [14].

Health service resource allocation therefore should focus on improving the quality of counselling interventions where they are poor but also on developing more effective interventions. Training for counsellors could be coupled with an investment in post-test care services where individuals at risk and those diagnosed with HIV can have their needs better met through the enhanced provision of support which may better address the multiple and intersecting barriers to change.

### Strengths and limitations

This study has some key strengths and limitations. A major strength is the meta-ethnographic method, which has allowed for the inclusion of a diverse range of in-depth, qualitative studies providing insights on multiple HTC delivery models embedded within broader contexts.

A limitation of this study is the age cut-off of 18. Adulthood can be understood as a problematic category, without a clear delineation in many cultures and with understandings rooted in parenthood, marriage, employment and change in social roles. Indeed the legal age of majority in ESA countries varies from 18 to 21 reflecting the contested nature of adulthood even within the region. The greatest reason for exclusion of potentially eligible studies was that they included participants under 18.

In addition, the studies which focused on evidence from HBVCT were from trials, meaning that the quality of the support provided could have been higher than that provided as standard of care outwith an intervention setting. It may also be the case that when these interventions are commissioned through health services that there is more incentive for providers to exert pressure on others to test, particularly if they are incentive based. More research is necessary to understand how these interventions function outside of trial settings.

Another more important limitation of this meta-ethnographic review is that unlike systematic reviews based on quantitative data which are replicable by design, meta-ethnographic approaches involve a significant amount of theoretical interpretation on behalf of the researcher and therefore the same conclusions may not be reached by others. Further, while data saturation was reached in studies related to VCT and PITC, it was not for HBVCT because we found fewer eligible studies investigating this intervention. We have made efforts to enhance this transparency by providing supporting information from our studies as an additional file.

Finally, while every effort has been made to ensure that this review is comprehensive, it is always possible that relevant studies or materials could have been missed in our searches and in our screening of potentially eligible material. This could potentially be compounded by ethnographic studies describing location only by country name rather than region, marking our searches perhaps less sensitive to these particular publications.

## Conclusions

HTC interventions in East and Southern Africa can be understood to have different goals depending on testing model. While VCT testing decisions were made on the individual level, HBVCT decisions were located in the family and community. PITC was characterised by high levels of coercion from clinical staff. Intensifying testing activity focusing on HBVCT models may be effective in reducing disparities in HTC uptake across the region in a way that is highly acceptable. All models have a role in a diverse mix of services targeting different groups within a population.

Counselling components in HTC interventions were effective in transmitting information about HIV and sexual risk, but were ineffective at addressing the broader personal circumstances preventing sexual behaviour change and modulating access to care. Our results suggest that counselling interventions are not useful for sexual behaviour change but remain a potential element in linking individuals who test positive into care in ESA.

## Supporting information

S1 Table Quality and relevance assessment form. (DOCX)

S2 Table Thematic construct table. (DOCX)

S1 Fig PRISMA checklist. (DOC)
