# Supplementary material for: Uptake contexts and perceived impacts of HIV testing and counselling among adults in East and Southern Africa: A meta-ethnographic review
Source: PLoS One. 2017 Feb 16;12(2):e0170588. doi: 10.1371/journal.pone.0170588 (PMC5313213; doi:10.1371/journal.pone.0170588)
Supplement: S1 Table — (DOCX) [file pone.0170588.s001.docx]

| **Authors** |  | **Date** |  |
| --- | --- | --- | --- |
| **Title** |  | | |
| **Methods (Q / M)** |  | **Journal** |  |
| **Quality** |  | **Relevance** |  |
| **Actual Impacts** |  | **Perceived** |  |

**Quality Scoring Guidelines:
3 – those that fulfil 6 or more indicators.
2 – Those that fulfil 4-5 indicators.
1 – Those that fulfil 1-4 indicators.**

| **Domain** | **Indicator** | **Present** |
| --- | --- | --- |
| **Research team and reflexivity** | **Are the researchers and their credentials made clear** |  |
| **Study design** | **Methodological orientation identified** |  |
|  | **Data collection methods identified** |  |
|  | **Sampling strategy and sample size identified** |  |
|  | **Description of sample provided** |  |
| **Analysis and findings** | **Clear description of analysis** |  |
| **Reporting** | **Clarity of research question** |  |

**Relevance**

| **3. Two HCTSSA primary study objectives directly addressed as primary objectives by study under review.** |  |
| --- | --- |
| **2. One primary HCTSSA objective part of main study question OR Two primary or secondary objectives from HCTSSA as secondary objectives of study.** |  |
| **1. HCTSSA objectives dealt with in limited way as part of analysis of data.** |  |
