# Supplementary material for: Uptake contexts and perceived impacts of HIV testing and counselling among adults in East and Southern Africa: A meta-ethnographic review
Source: PLoS One. 2017 Feb 16;12(2):e0170588. doi: 10.1371/journal.pone.0170588 (PMC5313213; doi:10.1371/journal.pone.0170588)
Supplement: S2 Table — (DOCX) [file pone.0170588.s002.docx]

| **Third Order Labels** | **Third Order Constructs** | **Second Order Constructs** | **First Order Constructs** |
| --- | --- | --- | --- |
| **Contexts of HTC decision making vary by model** | **VCT as a response to threatened identities from enduring illness.** | - *Discussions in the men’s groups revealed decision to postpone VCT until evidently sick. They acknowledged preferring to live in doubt of their status to living with the certainty of being infected as long as they were not symptomatic* (Nyanzi-Wakholi et al 2009). - *In recounting their experiences, many of those who tested or contemplating testing, described illnesses that significantly impaired their ability to work and/or to socialise with others in the public arenas, which created an urgent need to test.* (Siu et al 2014). - The first step on the HIV care pathway is the individuals’ decision to undergo HTC, and this was strongly influenced on one hand by their awareness of their ailing health and their knowledge of the potential benefits of ART treatment, and on the other by their fear of the consequences of being diagnosed HIV positive. (MacPherson et al 2012). - Therefore, individuals saw HTC as the first step towards regaining strength and avoiding an impending catastrophic social event, such as loss of employment or abandonment by a partner. Men also worried that, with increasingly outwardly obvious signs of failing health, they would be perceived to be less masculine and less respectable, with the assumption made that they could not provide for their family and not able to perform sexually. (MacPherson et al 2012). | - *Before I took the test I feared that I would commit suicide if I confirmed that I was HIV positive.* (Male, e-cohort) (Nyanzi-Wakholi et al 2009). - *I felt that I cannot continue living like this, I should know how my body is and then when I know how it is then I can know what to do next.* 22-year-old-man, single. (Sikasote et al 2011). - I have been ill for a long time, I have lost weight, my mouth has sores...all the symptoms of AIDS. People were even afraid of me and were talking behind my back, so I wanted to know for sure. (Male, 37 years) (Sethosa et al 2005). - Usually we wait for signs. We don’t go for check-ups until you get it. Yes, even HIV. Since most of the people don’t know to go for the HIV test because everybody feels okay and looks okay . . . And it’s very wrong. We must go for checkups . . . I have to feel ill before I’m going to the doctor . . . When I get ill, that’s when I am going to go for maybe HIV (test). (26-year-old female, HIV status unknown) (Daftary et al 2007). - *The illness has kept me coiled-up in bed and at home for weeks; I cannot work and [cannot] even go out to the centre to be with others. So I think where it has reached. I need to check.* Solomon (mid 30s) (Siu et al 2014). - *I am one of the people who does hard jobs: I stay in the sun the whole day building houses. From last year I was having stomach pains often, so I was just taking painkillers. Then I reached a stage of having fever regularly. So I was working one day and I felt weak and dizzy. I had heard that near my house there is VCT so I decided I have to go for a test* (Male participant, Chilomoni, 39 years). (Siu et al 2014) |
|  | **VCT as a means to restore order in response to past sexual risk.** | - Risk behaviour is one other reason that brings people to HIV testing. They will want to know if they have acquired the virus after engaging in risky behaviours (Sethosa et al 2005). - The testing process was facilitated by number of factors including sensitisation materials, the desire for peace of mind, the desire to regain control and the availability of antiretroviral drugs… (Sikasote et al 2011). - Many accounts revealed that the death of spouse or of a rival sexual partner increased the likelihood that a man might have contracted HIV and therefore needed to check (Siu et al 2014). | - *I started thinking of coming for the test because I noticed that my husband’s behaviour was not good. He has many women, so I am worried.* (44-year-old married woman) (Sikasote et al 2011). - *I wanted to know (if I am HIV positive) because I have slept with a guy I met at a party without using a condom.* (Sethosa et al 2005). - *A man raped me, I was so ashamed of myself afterwards, and I could not tell anybody about it, I just suffered alone. After some months I realized that I might be infected with HIV… I went to the hospital and for the first time.. I told the doctor, he then suggested that I do the tests.* (Female, 18 years) (Sethosa et al 2005). - It’s like you have committed a crime and then you are sentenced to death and then fortunately the President says “this person should be released”. It’s like you have already seen the point of death there and then you are told you are free now. You wouldn’t love to go to the same, to commit the same crime. 25-year-old man (single) 2^nd^ interview (Sikasote et al 2011). - The purpose of encouraging people to go for VCT, one of them is to make them have a free mind. Data management officer, male, focus group 1 (Sikasote et al 2005). - It was in 2008 when I lost her [wife]. She was very sick, only for one month and unfortunately passed away. After burying her, I got concerned about my health because before that I was not feeling well. I was feeling a lot of unexplained pain all over the body even before she died and so I decided to go for testing (Siu et al 2014). |
|  | **Encouragement from individuals in social or supportive networks in context of VCT, and PITC.** | - Men evaluated their risk of HIV infection and talked about testing with various categories of people, including spouses, parents and friends, in different contexts. However, it was mostly outside the domestic sphere and with peers that men reviewed their sexual histories and jointly discussed their risk of HIV infection (Siu et al 2014). - The majority of women who felt they had a choice in testing for HIV had thought about the issue prior coming to the clinic. Although many of these women discussed testing with family or partners before seeking antenat al care, they felt the final decision was theirs alone (Groves et al 2010). | - *A man raped me, I was so ashamed of myself afterwards, and I could not tell anybody about it, I just suffered alone. After some months I realized that I might be infected with HIV… I went to the hospital and for the first time.. I told the doctor, he then suggested that I do the tests.’* (Female, 18 years) (Sethosa et al 2005). - *Whenever I tell a friend that I am feeling pain all over the body, that friend just responds by saying, ‘why don’t you go for an HIV test?’ They [friends] can say ‘you have had many women and now you are pretending that you don’t know what is paining you!’ So it might be true [that I am infected] because when I look back, as a man…* (Siu et al 2014). - […] because he used to go there [for HIV treatment] and I said maybe I have the same problem like him. Also when you have a problem like this one, you tell a fellow man first because you know he can understand how it came about. You can talk about it with a friend and also talk about your movements [sexual contacts] (Siu et al 2014). - Man, I really urge you; do not miss this chance as you know what [sexual contacts] we have had. (Siu et al 2014). |
|  | **HBVCT testing decisions located on household and community spheres with pressure being applied based on social roles** | - The influences of the village headmen on individuals decisions to be tested was acknowledged, but important nuances emerged in the way the informants described this. (Jurgensen et al 2013). - To some extent, interactions and discussions among partners and other family members influenced individual decisions to test at home, though others stated that testing was a personal responsibility. The dynamics of discussion varied between respondents; patterns of influence often did not conform to the traditional hierarchy of many African families- for example we found sons and daughters to have encouraged their parents to take the test (Kyaddondo et al 2012). - Three potential sources of influence on an individuals’ decision to be tested emerged, namely: the partner, the headman and the counsellor (Jurgensen et al 2013). | - *Actually for me, my parents and siblings had a discussion on HIV testing and so my decision was influenced.* (Kyaddondo et al 2012). - *I asked my husband, because if I didn’t tell him he would have suspected that I misbehaved behind his back. So it was better to ask him, so that he knows what I am about to go through’ (Woman 37 years).* (Jurgensen et al 2013). - *Being the mother of the home, I influenced my son.* (Kyaddondo et al 2012). - *The headman [village leader] didn’t influence us, but encouraged us to choose whether to take part or not to take part. It’s up to an individual’s feeling.* (Jurgensen et al 2013). - Wives were expected to ask their husbands for permission to be tested. *‘I asked my husband, because if I didn’t tell him he would have suspected that I misbehaved behind his back. So it was better to ask him, so that he knows what I am about to go through’* (Woman 37 years). (Jurgensen et al 2013). |
|  | **Women in PITC are coerced into testing through the social roles of the health workers.** | - The perception of the pregnant women interviewed was that there was little opportunity to refuse HTC as the health workers are thought of as powerful, senior members of the community (MacPherson et al 2012) - A dominant message that pregnant women received at the clinic is that beingtest ed for HIV is the right thingto do for the health of the baby. Although that message may be accurate, it excludes any concern for the health and well-beingof the woman independent of her pregnancy. Perpetuation of such messages could further isolate and stigmatize the non-testers (Groves et al 2010). | - *You are forced to do it but in a helpful kind of way*. [A helpful kind of testing?] *Yes, they would tell you in the clinic and if you didn’t want to, you were free not to do it. The nurses in the clinic do not beg, if you don’t want to do it, you simply just don’t, but you would have a problem where you would be giving birth*’ (Anne, 28) (Groves et al 2010). - *I told myself but I was afraid. But because the nurses had told us that if you didn’t test you would not be assisted when giving birth because the same thing applies even if a person goes to a labor ward in a hospital. Then I was just scared . . . so scared. (Age 33, cohabitating)* (Groves et al 2010). |
|  | **Women’s identities as (future) mothers are leveraged by those in PITC** | - By simply evoking maternal/paternal responsibility to encourage uptake of HIV testing, couples were deprived of the right to consent and time and opportunity to reflect on the implications of HIV testing (Musheke et al 2013). - The perception of the pregnant women interviewed was that there was little opportunity to refuse HTC as health workers were seen as powerful, senior members of the community. There was also a perception that the government had decreed that all pregnant women should be tested for HIV, and this was interpreted to mean that there was no opportunity to refuse testing (MacPherson et al 2013). - During group counselling, observations revealed that the opt-out requirement was not explicitly articulated. Antenatal clinic staff often emphasised the bio-medical benefits of testing, including access to treatment and prevention of mother-to-child transmission (PMTCT) of HIV. Sometimes moral obligations were used to encourage uptake of HIV testing. | - *‘‘When you test you are not doing it yourself but for the baby’s safety. If you want to die you can do it on your own but you need to keep the baby safe’’.. and she said, ‘‘you know, there’s this mother whom we advised to test but she didn’t, it’s very painful to see her and her child sick.’’ (Age 30, cohabitating) (*Groves et al 2010) - *As of now, we give the first priority* [for testing] *to a woman who is pregnant. If the woman is expectant, she is expecting a baby… We want to protect the unborn child so that they should be born free from the virus.* Counsellor, female Ndirande, 23 years (MacPherson et al 2012). - One of the female informants compared her experience of the consent process during home-based VCT with her previous experience at the antenatal clinic: *I: But didn’t you go [for testing] at the PMTCT? R: I did, but that was a while ago, and at home it was of my own choice. It was not compulsory like at the antenatal clinic*. (Jurgensen et al 2013). |
| **Counselling interventions are inadequate to meet their stated aims in relation to sexual behaviour change and linkage to care due to the convergence of multiple barriers.** | **Inability of counselling to address broader patient circumstances affecting risk behaviour** | - Since VCT counsellor training had not prepared counsellors for working with MSM, most learned ‘‘on the job’’ about risk-taking behaviours, life events and the triggers for risk-taking behaviour (Taegtmeyer et al 2013). - Most respondents reported only being advised by their TASO counselors about condom use and not about reducing numbers of partners and/or frequency of sex. A few of the women wanted more guidance on negotiating condom use with partners. Women also reported less social support from both counselors and family and friends around reducing frequency of sex than did men (Lifshay et al 2009). - Pre-ART care infantilized patients and made them dependent on others | - *Sometimes they come and their risk is not their issue on that day. I feel that all I know is HIV and HIV related risk issues and that is all. I wish I had more knowledge and counselling skills in other areas*. (non-MSM counsellor on counselling MSM at risk of HIV) (Taegtmeyer et al 2013). - *So they gave me advice that I should avoid doing childish things like that. If I do, I should use condoms. But we are not youths we are elderly people.* Male participant, Ndirande, 45 years, did not initiate ART. (MacPherson et al 2012). - *Yesterday I have a client, a 32 years old man. Unfortunately the results was positive… I do the post-test counselling, while…he was shocked. And he, while he was shocked he just, there was the silence between me and him maybe for five to ten minutes. While we on the silence, there is a person outside. She want to come here on this room, because this room I used is the family planning room. So, now they want to do the family planning. Now, I suppose to reflect the feelings of this client, but I don’t have a chance, because there is a person outside*. (Rohleder & Swartz 2005). |
|  | **Sexual pleasure and linked condom preferences as barriers to risk reduction** | - Pain experienced by women when using condoms, continued sexual desire, partners’ desire for children, and assumptions about seroconcordance posed additional challenges for both consistent and non-consistent users (Lifshay et al 2009). - Many women and female partners disliked using condoms because they caused pain during intercourse (Lifshay et al 2009). | - *It reduces [pleasure] it makes the penis of a man become tight and small and he does not finish well.* (Jancita, 36 years, housewife) (Sarna et al 2009). - *Sometimes it burns her… it burn her in her private parts, so that is why ladies may prefer having unprotected sex…* (Consistent user, disclosed man)*.* (Lifshay et al 2012). - *He says ‘when I use them [condoms] I don’t feel any pleasure’ . . . I force him, I tell him that if he doesn’t want I leave him, we stay for a long time until in the end he accepts and uses it . . . then he complains the whole time, he quarrels. At times I try to dodge him; I dodge and dodge until I get tired . . .* (Zainab, 36 years, housewife) (Sarna et al 2009). |
|  | **Feelings of inevitability surrounding HIV transmission as constraining choice** | - In long relationships between discordant partners, a false sense of immunity interwoven with expressions of fatalism also obstructed condom use. (Sarna et al 2009). | - *It is because he thinks that even if he is negative now, he will eventually become positive, so he wants sex without a condom.* (43 year old HIV+ women in serodiscordant relationship) (Emusu et al 2009). - *She told me that we have been having sex all these years and if it is death then I am ready to die with you; but I was counselled on the importance of using condoms and I told her it is important we use condoms I feel like sleeping with her but sometimes I just restrain myself.’* (Gideon, 34 years, gardener). (Sarna et al 2009). |
|  | **Issues relating to gender roles within relationships modulated by socio-economic factors** | - The suspicion of infidelity with subsequent apportioning of blame as to who brought the HIV infection into the relationship played an important role in propagating sexual violence against women. Being younger and often physically weaker, as well as being economically dependent on the male partners hindered the women’s ability to resist sexual advances of partners perceived to be at high risk of transmitting or getting infected with HIV. (Emusu et al 2009) - Nonconsistent users had to contend with gender-power imbalances and male partners’ lack of pleasure when using condoms.(Lifshay et al 2009) | - *He told me that if you do not want sex without a condom, then you will go back to your parents and I will find another woman to marry. So since I am scared of losing my marriage, I just give him ‘live’ sex. What can I do because when I tell him about using condoms, he refuses, so what I can I do, apart from re-infecting him?.... How am I going to look after myself and my children?* (29 year old woman in sero-concordant relationship) (Musheke et al 2013) - *Because income is low, someone is forced to have sex… Sometimes I need soap, sugar, and transport to (the clinic)… I am forced to have sex with him because if I refuse, he will not provide; maybe he will disappear forever*. (Non-consistent condom user, disclosed woman) (Lifshay et al 2009). |
|  | **Cultural beliefs held about HIV and HIV transmission.** | - Many participants reported that after they had tested for HIV and received HIV education, they requested that their partners also test. Many refused, claiming that if the woman tested negative, then he was also negative as they were having unprotected sex. (Shamu et al 2012) | - *Some men have lost their wives to HIV/AIDS but they lie to us that they were found HIV negative when they tested. This is not true.* (Male, 35 years old, Nyakiyumbu FGD). (Bwambale et al 2008) - *I asked my partner to go for a test but he refused. He said he does not have the disease saying that since I didn’t have it meant that he didn’t have it as well. I told him to go for tests. I even refused to sleep with him… In the end I gave in because he was not going for the tests anyway (FGD PNC Women, Facility A).* (Shamu et al 2012) - *When suspicious of HIV, men do not usually test through the needle but through having a baby, knowing that the wife will be tested at the clinic…* (HIV Counsellor, Facility E). (Shamu et al 2012). |
|  | **Tensions between notions of safety and traditional gender roles.** | - For some couples, having unprotected sex was construed as sine qua non for consummating marital relationships while condom use was synonymous with having sex with a non-regular sexual partner - with no emotional intimacy attached (Musheke et al 2013). - Some participants use condoms for the purposes of both family planning and prevention of HIV transmission. They mostly reported consistent condom use and generally were not using other family planning methods. By contrast, others expressed a desire for children and this was an important reason for not using condoms. This desire appears to supersede concerns about the risk of HIV transmission (Sarna et al 2009). | - *She kept asking me how we would get a child if we keep on using condoms . . . initially we agreed to use but later on she turned her back and says that I am mistreating her . . . this puts me in a tricky situation . . . sometimes I think that I should let her go out with other men…. My wife sees no use of using a condom because she wants to have a baby.* (Gideon, 34 years, gardener) (Sarna et al 2009). |
